# The Impact of Acute Heart Failure on Frailty Degree and Outcomes in Elderly Patients with Severe Aortic Stenosis and Chronic Heart Failure with Preserved Ejection Fraction

**DOI:** 10.3390/jcdd11050150

**Published:** 2024-05-14

**Authors:** Augusto Esposito, Ilenia Foffa, Cecilia Vecoli, Luca Bastiani, Sergio Berti, Annamaria Mazzone

**Affiliations:** 1 Fondazione Toscana Gabriele Monasterio, 54100 Massa, Italy; augustoesposito1990@gmail.com (A.E.); cecilia.vecoli@cnr.it (C.V.); luca.bastiani@cnr.it (L.B.); sergio.berti@ftgm.it (S.B.); mazzone@ftgm.it (A.M.); 2 CNR Institute of Clinical Physiology, 54100 Massa, Italy

**Keywords:** acute heart failure, multidisciplinary approach, frailty, elderly patients, severe aortic stenosis, hospitalization, mortality

## Abstract

Frailty degree plays a critical role in the decision-making and outcomes of elderly patients with severe aortic stenosis (AS). Acute heart failure (AHF) results in a severely worse clinical hemodynamic status in this population. This study aimed to evaluate the impact of AHF on frailty degree and outcomes in older patients referred for tailored interventional treatment due to AS. A total of 109 patients (68% female; mean age 83.3 ± 5.4), evaluated by a multidisciplinary path for “frailty-based management” of valve disease, were divided into two groups, one with (AHF+) and one without AHF (AHF-) and preserved ejection fraction (mean value EF: 57.4 ± 8.6). AHF occurred a mean value of 55 days before geriatric, clinical, and surgical assessment. A follow-up for all-cause mortality and readmission was conducted at 20 months. AHF+ patients showed a higher frequency of advanced frailty (53.3% vs. 46.7%, respectively), rehospitalization (35.5% vs. 12.8; *p* = 0.007), and death (41.9% vs. 12.8%; *p* < 0.001). In stepwise logistic regression analysis, AHF emerged as an independent risk factor for advanced frailty (OR: 3.8 CI 1.3–10.7; *p* = 0.01) and hospital readmission (OR: 3.6 CI 1.1–11.6; *p* = 0.03). In addition, preceding AHF was an independent determinant associated with a higher risk of mortality (HR 2.65; CI 95% 1.04–6.74; *p*-value 0.04). AHF is independently associated with advanced frailty and poor outcomes in elderly patients with severe AS. So, this population needs careful clinical and geriatric monitoring and the implementation of interventional therapy for AS in the early stages of frailty to avoid the occurrence of AHF and poor outcomes.

## 1. Background

Degenerative aortic stenosis (AS) is a valve disease whose prevalence has grown as the population grows older [1]. The progression of AS is accompanied by many comorbidities, such as coronary artery disease, arterial hypertension, diabetes, kidney disease, and other valvular diseases that contribute to the development of chronic heart failure (HF) with preserved or reduced ejection fraction (HFpEF or HFrEF) [2,3]. 

AS and HF are two common causes of mortality in the elderly and their coexistence increases the rates of mortality and morbidity [2]. The concomitance of severe AS and HF characterizes an important and high-risk group of patients who are referred for transcatheter aortic valve replacement (TAVR) as an alternative to surgical intervention. Elderly patients with AS and HF benefit from TAVR with respect to symptom alleviation. In patients with HFpEF, TAVR leads to an identical, favorable post-procedural prognosis that is significantly better than that of patients with HFrEF, who remain a high-risk population [3]. Randomized trials have demonstrated improvements in survival and symptoms after TAVR compared to medical therapy (MT) in the elderly population, but a percentage of treated patients do not benefit from TAVR, either due to death or to a lack of improvement in quality of life. Frailty, a progressive geriatric syndrome, has been confirmed as an important prognostic factor [4] of mortality post TAVR, and is emerging as a clinical decision-making parameter in the management of AS in elderly patients [5]. Furthermore, frailty and HF share a common pathophysiological background, including inflammation, malnutrition, and sarcopenia, and are strongly associated with each other [6]. Clinical monitoring of aortic stenosis progression, heart failure, and frailty degree may be crucial to identify the right time for interventional treatment of AS in elderly patients. Conditions such as frailty and geriatric syndromes are usually measured in older patients with chronic heart failure (CHF) [7,8], while limited research has been conducted on patients with acute heart failure (AHF). However, evidence suggests that frailty is associated with worse prognoses in elderly AHF patients [9,10].

Thus, Reeves et al. showed that older acute decompensated HF patients were frequently frail, with widespread impairments in physical function, cognition, mood, and quality of life, conditions that may contribute to their persistently poor outcomes [11]. Interestingly, frailty was an independent marker of early congestive heart failure admission with a powerful association with moderate-severe degenerative aortic stenosis in geriatric patients [12]. In this clinical study, we aimed to retrospectively evaluate the burden of AHF on frailty degree in older patients with severe AS and HFpEF, selected for tailored treatment by a comprehensive pre-TAVR assessment [5]. In addition, in this elderly population, we aimed to analyze the impact of a preceding AHF event on all-cause mortality and hospital readmission at medium-term follow-up.

## 2. Methods

### 2.1. Study Population

We retrospectively analyzed 109 patients (68% female; mean age 83.3 ± 5.4) at Ospedale del Cuore FTGM in Massa, Italy, who, from 1 March 2016 to 30 March 2020, were randomly recruited among elderly symptomatic AS patients for multidimensional assessment, conducted by a multidisciplinary path characterized by an integrated clinical, surgical, and geriatric risk assessment for “frailty-based management”, and for tailored treatment of valve disease, including surgical aortic valve replacement (SAVR), TAVR, balloon aortic valvuloplasty (BAV), or medical therapy (MT), as described in our previous paper [5]. Patients with acute heart failure and hemodynamic shock were excluded at the time of clinical evaluation at the day service center and interventional procedures were performed within 60 days after the baseline examination, based on urgency. The median follow-up period was 20 months, and no patients were lost to follow-up. 

The diagnosis of CHFpEF was based on the criteria of left ventricular ejection fraction (LVEF) ≥ 50% on a transthoracic cardiac echocardiographic (TTE) test on admission and a value of brain natriuretic peptide (BNP) ≥ 100 pg/mL, according to the clinical guidelines [13]. The study population was divided into two groups: patients with a preceding AHF event (AHF+) and patients without a preceding AHF event and preserved ejection fraction (AHF-). An AHF event was considered if it occurred in the period of roughly 55 days before the multidimensional assessment. Patients with acute decompensated heart failure at the time of the multidisciplinary evaluation were excluded. The two groups of patients underwent a clinical, laboratory, and multi-geriatric assessment (MGA) to evaluate comorbidities, disability, cognitive function, depression, and nutritional status, using the following validated indices: Charlson Index (CI), Basic Activities of Daily Living (BADL), Instrumental Activities of Daily Living (IADL) to test for disability, Mini Mental State Examination for cognitive function evaluation (MMSE at ≤18 points, cognitive impairment), Geriatric Depression Score for mood disorder (GDS at ≥5 points, depression), and Mini Nutritional Assessment (MNA ≤ 8 points) for nutritional status. Mortality and hospital readmissions were registered at 20 months’ follow-up. This study was approved by the local Ethics Committee (No. 22239).

### 2.2. Statistical Analysis

Continuous variables were reported as mean ± SD or median (IQR) depending on normality. Categoric variables were expressed as absolute numbers (*n*) or percentages (%). Normality was assessed using the Kolmogorov–Smirnov test. Comparisons between the groups were carried out using Student’s *t*-test for continuous data and the chi-square test for categoric variables. Multivariate logistic regression analysis was performed to explore risk and protective factors with respect to frailty and patient rehospitalization. Furthermore, a multivariate Cox regression analysis with stepwise backward conditional elimination of non-significant factors was used to explore a model predicting patient survival. All analyses were performed using the IBM Statistical Package for Social Sciences (SPSS, version 23, Chicago 2013) and a two-sided *p*-value < 0.05 was considered statistically significant.

## 3. Results

One hundred and nine elderly patients with symptomatic severe AS were divided into two groups: patients with AHF (AHF+) and patients without AHF and preserved ejection fraction (AHF-). Clinical, echocardiographic, laboratory, surgical, frailty status, and outcome data are detailed in Table 1.

The mean age of the patients was 83.3 ± 5.5, and 68% were female, without significant differences between the groups. We found 31 patients (28%) in whom a previous acute decompensated HF event had occurred a mean value of 55 days before the multidimensional assessment; in particular, 72% of these patients had received HF hospitalization. Considering traditional risk factors, only diabetes was significantly more frequent in the AHF + group, which also showed a higher NYHA class (III-IV) (*p* = 0.0001). 

Regarding transthoracic echocardiographic imaging, AHF+ patients had a lower EF (*p* = 0.0001), higher PAPs (*p* = 0.001), lower mAVG (*p* = 0.013), and higher STS scores (*p* = 0.023). Regarding the laboratory parameters, we found higher values for BNP (*p* = 0.0001), creatinine (*p* = 0.0001), uricemia (*p* = 0.02), C-reactive protein (CRP) (*p* = 0.008), Troponin I (*p* = 0.01), and neutrophils (*p* = 0.003) in AHF+ patients, while albumin (*p* = 0.014) and Creatine phosphokinase (CPK) (*p* = 0.004) levels were lower.

The prevalence of geriatric impairments according to physical frailty status is shown in Table 2. 

In the group of AHF+ patients, we found a higher malnutrition status (*p* = 0.0002), more comorbidities (*p* = 0.002), and more frequent depression (*p* = 0.007), as well as a higher frequency of sarcopenic patients (*p* = 0.001) and a lower IADL score (*p* = 0.002). 

### Acute Heart Failure and the Management of Elderly Patients with Severe AS

**Figure 1:**
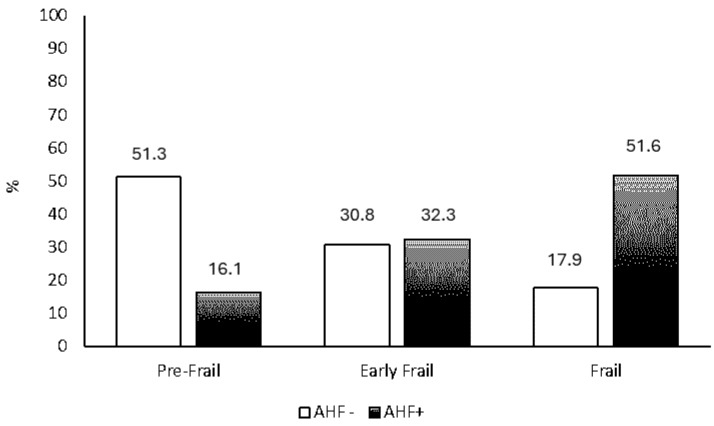
Frequency of physical frailty degree in patients with (AHF+) or without (AHF-) acute heart failure.

The degree of physical frailty, according to the Fried criteria score, was significantly higher in AHF+ patients (*p* = 0.0001). In particular, AHF+ patients had a higher frequency of advanced frailty with respect to AHF- (53.3% vs. 46.7%, respectively) and a lower frequency of pre/early frail (11.1% vs. 29.4%) (Figure 1).

According to the Fried frailty score, 48.4% of AHF+ patients were treated by BAV, 32.3% by TAVR, 19.4% by MT, and none by SAVR.

Moreover, in patients with an event of AHF, we found a higher frequency of rehospitalization (35.5% vs. 12.8; *p* = 0.007) and death (41.9% vs. 12.8%; *p* < 0.001). Regarding readmissions, in the group of frail patients, we found a higher frequency of readmission in patients with AHF (64% vs. 36%); in particular, these patients were treated by medical therapy (*n* = 3) or BAV (*n* = 4).

In stepwise logistic regression analysis, adjusted for age and gender, we found that STS scores, BADL, and Charlson scores were not statistically significant, while previous AHF event was an independent risk factor for advanced frailty (OR: 3.8, CI 1.3–10.7; *p* = 0.01) (Table 3).

In particular, previous AHF event was also an independent risk factor for rehospitalization (OR: 3.6 CI 1.1–11.6; *p* = 0.03) (Table 4).

**Figure 2:**
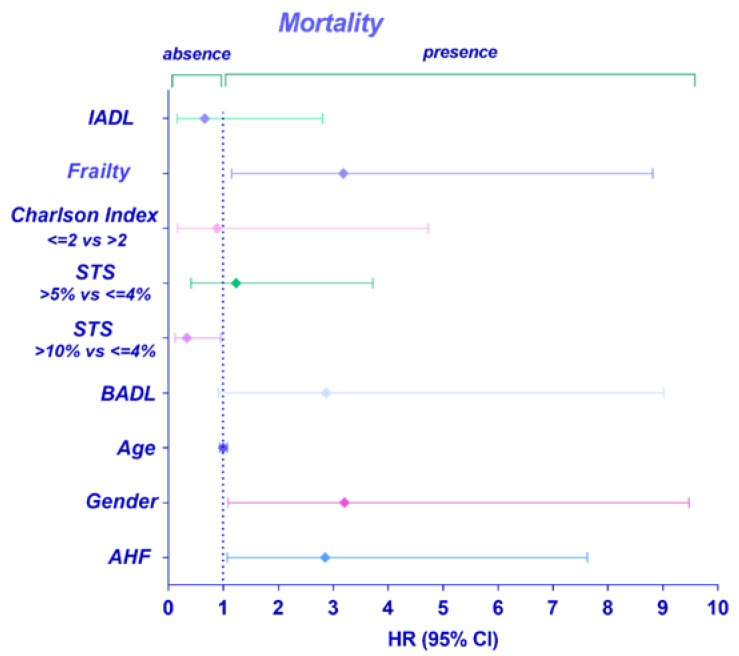
Cox proportional hazard regression analysis of risk of all-cause mortality in elderly AHF+ patients.

Moreover, the multivariate Cox regression model (adjusted for age, gender, STS score, BADL, IADL, Charlson score, and frailty) showed that a preceding event of AHF was an independent determinant associated with a higher risk of mortality during the follow-up period (HR 2.8; CI 95% 1.07–7.6; *p*-value 0.037) (Figure 2).

## 4. Discussion

In this study, for the first time, we demonstrated the burden of an acute event of HF on frailty degree and adverse outcomes in terms of mortality and rehospitalization in an elderly population with severe AS. Our data suggest that the concomitance of severe AS and HF characterizes a significant and high-risk group of patients who need correct management to improve prognoses and in whom the risk–benefit ratio of invasive diagnostic and therapeutic procedures must be considered.

It is well known that severe, symptomatic aortic stenosis may cause heart failure, acute myocardial infarction, or syncope; however, data on the occurrence of such events before transcatheter aortic valve replacement (TAVR) and their impact on subsequent outcomes are limited [14]. An improvement in existing knowledge may help in the clinical management and identification of patients in need of pre- and post-TAVR optimization to increase the likelihood of improved outcomes. In fact, the timing of intervention is crucial for improving the quality of life or even prolonging patients’ life expectancy versus procedural risk. 

### 4.1. Clinical, Surgical, and Geriatric Features in AHF Patients

Literature data report that HFpEF is the predominant disease type in the HF population, mainly among the elderly, and that HFpEF patients have a higher burden of comorbidities than HFrEF patients [15]. Accordingly, we highlighted that a previous AHF event in an elderly patient with severe AS and HFpEF worsens their clinical, surgical, and functional features. In particular, patients with preceding AHF events have a worsened systolic function, switching from HFpEF to HFmEF. These changes are due to the increase in afterload caused by the progressive narrowing of the aortic valve, left ventricular remodeling, increased mitral valve regurgitation, and right ventricular damage with higher PAPs, highlighting a worse NYHA class evolution [16]. Among the cardiovascular risk factors, as reported in the literature [17], diabetes appears to be associated with episodes of AHF, and regarding their surgical evaluation, AHF patients have higher STS scores. Moreover, considering the parameters used for the MGA, patients with AHF showed higher levels of comorbidities and used a higher number of drugs as pharmacotherapy. In addition, they were significantly more affected by depression, with worse, but not significant, cognitive impairment, and presented a higher functional level of disability, according to IADL scores [11]. Malnutrition and sarcopenia are very common in frail patients with heart failure [18], and also in this population, we identified the coexistence of poor nutritional status (according to the MNA) and of significant sarcopenia (as assessed by the hand grip test) [19]. The presence of malnutrition status and sarcopenia is also supported by lower values of proteins as well as of serum albumin and CPK and by a chronic status of inflammation, expressed by a significant increment in CRP and neutrophil values [20]. The compromised cardiovascular picture post AHF is confirmed by increased levels of BNP, troponin I, and creatinine in these patients. 

### 4.2. Frailty Degree in AHF Patients

In recent years, as research on frailty has increased, a high incidence of frailty has been identified in HF patients, increasing the risk of adverse outcomes such as mortality or hospitalization [21]. In fact, older patients with an acute decompensated HF event are frequently frail, with a concomitance of severe and widespread impairments in physical function and cognition. This multi-organ nature of impairment, due also to hospitalization, increases their susceptibility to frailty and their vulnerability to adverse clinical events [11,22,23]. Our data express a significant association between an advanced level of physical frailty and the occurrence of a previous episode of acute decompensated heart failure. Conversely, patients without a previous episode of AHF had early and reversible stages of frailty at the time of our multidisciplinary evaluation. To confirm the burden of an acute heart failure event on frailty, our stepwise logistic regression analysis showed that an acute HF event was an independent risk factor for an advanced degree of frailty. These original data may be useful in the clinical evaluation of elderly patients to establish the correct timing of treatment. In particular, it is important to avoid the occurrence of an acute decompensated heart failure event, as it may lead to clinical and functional worsening and could make interventional treatment of aortic stenosis futile. In fact, in this study, patients that had previously had an AHF event and showed advanced levels of physical frailty were treated by BAV and medical therapy. These tailored treatments were also suggested in the presence of chronic renal failure and comorbidities. 

### 4.3. AHF, Frailty and Outcomes

Frailty and HF are associated with increased adverse outcomes, such as hospitalizations and mortality, especially in older patients [24].

**Figure 3:**
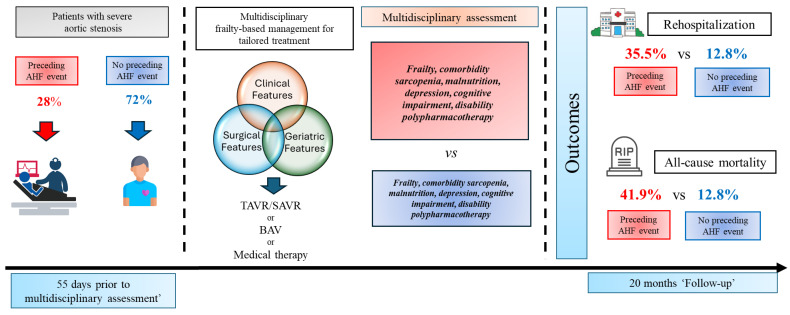
Schematic representation of study protocol and the major results.

When an acute decompensated heart failure event occurs in a patient with HFpEF, it proves responsible for subsequently worse levels of comorbidity, disability, and frailty, even despite medical treatment for hemodynamic recovery during hospitalization. In particular, AHF seems to also be responsible for worse prognoses in terms of all-cause mortality and recurrence of rehospitalization. Frail patients treated by BAV seem to be the most subjected to new hospitalizations for acute decompensated HF [25]. This procedure needs to be repeated in selected cases, and mortality after repeated BAV is high due to its unfavorable risk profile. In particular, there is a significant rate of non-cardiac deaths in those patients, which may be related to multiple comorbidities leading to the denial of definitive treatment in this group [25]. Other recent studies showed that AHF readmission is common, affecting almost one-sixth of TAVR recipients within 1 year of the procedure, and late and multiple HF readmissions are associated with an increased risk of long-term all-cause mortality. In addition, among baseline comorbidities, previous acute HF might predict HF readmission [26,27]. According to these data, in AHF patients, we found both an increased frequency of all-cause mortality and rehospitalization during two years of follow-up, and that an acute event of HF proved to be an independent determinant associated with a higher risk of mortality and rehospitalization. Figure 3 shows a schematic representation of the study protocol and the major results.

Therefore, in an elderly population with degenerative aortic valve disease, strategies for preventing and better managing HF before and after TAVR are necessary to improve outcomes. In particular, based on the evidence in our study, the occurrence of frailty and AHF characterizes a significant and high-risk group of patients, and in this population, it is important to perform a multidimensional assessment for tailored treatment. Thus, it is important to enhance risk stratification using novel management strategies in order to improve outcomes and to reduce the burden of an acute event of HF in this vulnerable population. In particular, novel prospective studies are necessary to design an accurate assessment for the older population and to evaluate the correct timing for TAVR/SAVR prior to the development of severe symptomatic AS, high frailty levels, and AHF events. This approach could improve the clinical and functional status as well as the mortality and rehospitalization rates and the quality of life of the patient.

### 4.4. Study Limitations

This study has several limitations, including its retrospective nature. Moreover, it is a monocentric study performed on a relatively small number of patients. However, despite these limitations, the study of this well-characterized population has allowed us to reach some valuable conclusions that pave the way for further investigations in larger populations.

## 5. Conclusions

In our study, we highlight for the first time that acute decompensated HF events are independently associated with an advanced frailty degree and poor outcomes in elderly patients with severe symptomatic AS and HFpEF. This older population, which is expected to constantly increase, needs careful clinical and geriatric monitoring to implement an effective interventional therapy for AS, especially in the early and reversible stages of frailty, avoiding the occurrence of AHF events, which have a negative impact on frailty and outcomes. So, it is important to identify elderly AS patients who could benefit from TAVR/SAVR treatment. Therefore, in this elderly population, the careful monitoring of many clinical/geriatric, laboratory, and imaging parameters correlated to aortic valve stenosis progression and chronic heart failure may help in the identification of the best time to implement an effective and not unsuccessful aortic interventional therapy. 

## Figures and Tables

**Table 1:** Baseline characteristics of study population.

*Variables*	All Patients (*n* = 109)	AHF+ (*n* = 31)	AHF- (*n* = 78)	*p*-Value
Age (yrs)	83.3 ± 5.5	83.9 ± 6.3	83.1 ± 5.1	0.490
Female, *n*	74 (68)	23 (74.2)	51 (65.4)	0.374
***Comorbidities***				
Hypertension	97 (89)	28 (90.3)	69 (88.5)	0.779
Hypercholesterolemia	80 (73.4)	19 (61.3)	61 (78.2)	0.071
Diabetes	39 (35.8)	17 (54.8)	22 (28.2)	**0.009**
Smoking	29 (26.6)	8 (25.8)	21 (26.9)	0.655
COPD	46 (42.2)	17 (54.8)	29 (37.2)	0.092
Previous AMI	18 (16.5)	5 (16.1)	13 (16.1)	0.946
Previous stroke	15 (13.8)	4 (12.9)	11 (14.1)	0.870
Chronic heart failure	68%	28%	40%	
***NYHA classes***				
I–II	70 (64.2)	7 (22.6)	63 (80.8)	**0.0001**
III–IV	39 (35.8)	24 (77.4)	16 (19.2)	
Angina	33 (30.3)	10 (32.3)	23 (29.5)	0.776
***Echo parameters***				
PAPs	46.7 ± 11.3	52.5 ± 13.2	44.5 ± 9.6	**0.001**
EF, %	57.4 ± 8.6	50.9 ± 10.8	60.0 ± 5.9	**0.0001**
mAVG, mmHg	44.1 ± 12.3	39.5 ± 11.3	45.9 ± 12.2	**0.013**
STS score	4.45 (2.7–6.1)	5.4 (4.3–10)	4.1 (2.3–5.0)	**0.023**
***Laboratory parameters***				
BNP, pg/mL	281 (134–588.2)	528 (343–1182)	195 (120–369)	**0.0001**
Creatinine, mg/dL	1.09 (0.9–1.4)	1.4 (1.1–2.0)	0.98 (0.86–1.21)	**0.0001**
Albumin, g/L	4.0 ± 0.4	3.83 ± 0.59	4.10 ± 0.36	**0.014**
Uricemia, mg/dL	6.1 ± 1.8	6.7 ± 2.2	5.7 ± 1.6	**0.02**
Creatine phosphokinase (CPK), IU/L	68.6 ± 36.4	52.5 ± 24.3	74.9 ± 24.3	**0.004**
Troponin I, µg/L	0.03 ± 0.03	0.04	0.02	**0.01**
Neutrophils, n/µL	5069.5 ± 1987	5934.2 ± 2484	4725.8 ± 1647	**0.003**
C-reactive protein (CRP), mg/dL	0.74 ± 1.5	1.3 ± 2.4	0.5 ± 0.78	**0.008**
***Aortic valve treatment***				
SAVR	8 (7.3)	0 (0.0)	8 (10.3)	**0.0001**
TAVR	59 (54.1)	10 (32.3)	49 (62.8)	
BAV	27 (24.8)	15 (48.4)	12 (15.4)	
MT	15 (13.8)	6 (19.4)	9 (11.5)	
***Physical frailty***				**<0.0001**
Frail	30 (27.5)	16 (51.6)	14 (17.9)	
Early frail	34 (31.2)	10 (32.3)	24 (30.8)	
Pre-frail	45 (41.3)	5 (16.1)	40 (51.3)	
***Outcomes***				
Rehospitalization	21 (19.3)	11 (35.5)	10 (12.8)	**0.007**
Death	23 (21.1)	13 (41.9)	10 (12.8)	**<0.001**

BNP: brain natriuretic peptide; COPD: chronic obstructive pulmonary disease; AMI: acute myocardial infarction; NYHA: New York Heart Association; PAPs: systolic pulmonary artery pressure; EF: ejection fraction; mAVG: mean aortic valve gradient; STS: Society of Thoracic Surgeons; SAVR: surgical aortic valve replacement; TAVR: transcatheter aortic valve replacement; BAV: balloon aortic valve; MT: medical therapy. Bold means the statistically significant.

**Table 2:** Geriatric assessment according to frailty status.

*Variables*	AHF+ Patients	AHF- Patients	*p*
**Total number of drugs (mean ± SD)**	7.6 ± 1.8	6.4 ± 2.6	**0.02**
**Charlson Comorbidity Index, CCI**	5.5 ± 2.3	4.5 ± 2.1	**0.002**
**Nutrition status, MNA pts**	9.1 ± 3	10.9 ± 1.8	**0.0002**
**Depressive symptoms, GDS pts**	3.2 ± 3.1	5.1 ± 3.5	**0.007**
**Cognitive status, MMSE pts**	23.2 ± 5.4	25.2 ± 4.7	0.06
**Sarcopenia, *n* (%)**	23 (74)	31 (39)	**0.001**
***Disability***			
**IADL**	5 ± 2.4	6.5 ± 2.1	**0.002**
**BADL**	4.8 ± 1.7	5.3 ± 1.4	0.1

Values are mean ± SD, *n* (%). CCI: Charlson Comorbidity Index (score range, 1–12); MNA: Mini Nutritional Assessment (score range, 1–14); GDS: Geriatric Depression Scale (score range, 0–13); MMSE: Mini Mental State Examination (score range, 1–30); BADL: Basic Activities of Daily Living, (score range, 0–6); IADL: Instrumental Activities of Daily Living (score range, 0–8). Bold means the statistically significant.

**Table 3:** Logistic regression analysis for advanced frailty.

*Variables*	OR	(95% CI)		*p*-Value
***AHF***	3.837	1.373	10.721	0.010
***Gender***	0.434	0.139	1.360	0.152
***Age***	0.977	0.895	1.066	0.609
***BADL***	2.629	0.954	7.237	0.061
***STS > 5% vs. <=4%***	0.898	0.299	2.695	0.848
***STS > 10% vs. <=4%***	0.615	0.151	2.491	0.496
***Charlson Index Score (<=2/>2)***	0.142	0.016	1.208	0.074

AHF: acute heart failure; BADL: Basic Activities of Daily Living (score range, 0–6); STS: Society of Thoracic Surgeons.

**Table 4:** Logistic regression analysis for rehospitalization.

*Variables*	OR	(95% CI)		*p*-Value
***AHF***	3.630	1.127	11.689	0.031
***Gender***	2.164	0.645	7.262	0.211
***Age***	0.965	0.878	1.060	0.457
***BADL***	3.605	1.092	11.903	0.035
***Charlson Index Score (<=2/>2)***	1.887	0.385	9.248	0.434
***Frailty***	1.917	0.579	6.343	0.286

AHF: acute heart failure; BADL: Basic Activities of Daily Living (score range, 0–6).

## Data Availability

The dataset of this study is available from the corresponding author upon reasonable request.
